# Associations between Screen-Based Activities, Physical Activity, and Dietary Habits in Mexican Schoolchildren

**DOI:** 10.3390/ijerph18136788

**Published:** 2021-06-24

**Authors:** Erica G. Soltero, Alejandra Jáuregui, Edith Hernandez, Simón Barquera, Edtna Jáuregui, Juan Ricardo López-Taylor, Luis Ortiz-Hernández, Lucie Lévesque, Rebecca E. Lee

**Affiliations:** 1 USDA/ARS Children’s Nutrition Research Center, Department of Pediatrics, Baylor College of Medicine, 1100 Bates Avenue, Houston, TX 77030, USA; Edith.Hernandez@bcm.edu; 2 Centro de Investigación en Nutrición y Salud, Instituto Nacional de Salud Pública, Universidad No. 655 Colonia Santa María Ahuacatitlán, Cerrada Los Pinos y Caminera, Cuernavaca 62100, Mexico; alejandra.jauregui@insp.mx (A.J.); sbarquera@insp.mx (S.B.); 3 Instituto de Ciencias Aplicadas a la Actividad Física y Deporte, Centro Universitario de Ciencias de la Salud, Universidad de Guadalajara, Independencia Oriente, Guadalajara 44340, Mexico; edtna.jauregui@hotmail.com (E.J.); taylor@cucs.udg.mx (J.R.L.-T.); 4 Departamento Medicina Preventiva, Secretaria de Salud, Dr. Baeza Alzaga #107, Colonia Centro, Mexico City 44100, Mexico; 5 Departamento de Atención a la Salud, Universidad Autónoma Metropolitana Unidad, Calzada del Hueso 1100, Col. Villa Quietud, Delegación Coyoacán, Xochimilco, Distrito Federal 04960, Mexico; lortiz@correo.xoc.uam.mx; 6 School of Kinesiology & Health Studies, Queen’s University, 28 Division Street, Kingston, ON K7L3N6, Canada; levesqul@queensu.ca; 7 Center for Health Promotion and Disease Prevention, Edson College of Nursing and Health Innovation, Arizona State University, 550 N. 3rd Street, Phoenix, AZ 77092, USA; releephd@yahoo.com

**Keywords:** screen time, physical activity, diet, children, Hispanic

## Abstract

Screen-based activities are associated with increased risk of obesity and contribute to physical inactivity and poor dietary habits. The primary aim of this study was to examine the associations among screen-based activities, physical activity, and dietary habits in school-aged children in Guadalajara, Puerto Vallarta, and Mexico City, Mexico. The secondary aim was to examine these associations across sex. The School Physical Activity and Nutrition survey was used to assess screen-based activities (TV watching, video game use, computer use), physical activity, and dietary habits. Organized activity/sports participation, unhealthy dietary habits, and household income were correlated with screen-based activities. While TV watching was associated with decreased participation in organized activity/sports participation, computer and video game use was associated with increased organized activity/sports participation. Boys engaged in more TV watching and video game use compared to girls. All screen-based activities were associated with age among boys; whereas video game and computer use were associated with higher income among girls. These findings suggest a need for sex- and age-specific strategies that acknowledge the differential use of screen-based activities across sex and age. Future research should continue to identify underlying correlates linking screen-based activities with health behaviors to inform strategies to reduce screen-time in Mexican children.

## 1. Introduction

Pediatric obesity is a critical public health challenge in Mexico; the prevalence of obesity among Mexican children has reached 35.8% among Mexican girls and 34.1% among Mexican boys [1]. An extensive body of literature has demonstrated that screen time significantly contributes to pediatric obesity and obesity-related disease risk factors including hypertension and insulin resistance [2,3]. As technology has become a ubiquitous part of everyday life, today’s youth are exposed to technology at a younger age and are the largest users of digital devices [4]. Nearly 30% of Mexican children own a smartphone, 56% access the internet on a daily basis, the adoption rate of video games is estimated at 90%, and about 50% have access to computers and tablets, respectively [5,6]. Global screen time guidelines recommend that children should be limited to less than or equal to 2 h of screen time per day [7,8]. However, the most recent National Health and Nutrition Survey reported that 77.3% of Mexican children exceeded the 2-h screen time limit [9]. Given that screen time significantly contributes to obesity, there is an urgent need to increase our understanding of how screen time impacts obesity-related diet and physical activity behaviors among Mexican children to inform intervention strategies focused on reducing screen time.

The relationship between screen time and obesity is driven in part by reduced physical activity and overall increased energy intake [10,11]. Screen time significantly contributes to overall sedentariness [12,13] and reduces energy expenditure, physical fitness, and muscle strength [14,15], as it displaces time that may have otherwise be spent in physical activities [13,16,17]. Screen time also impacts diet as it is associated with increased snacking, which typically consists of calorically dense, high fat, “junk” foods [18,19,20,21,22]. In addition, many food advertisements during children’s programming focus on unhealthy foods, which has been shown to have a negative impact on diet in children [17,23]. Despite this growing body of literature, the evidence on how screen time impacts diet and physical activity behaviors in Mexican children remains limited [24].

It has been hypothesized that some screen-based activities may be more deleterious compared to others, suggesting that different types of screen-based activities may differentially impact diet and physical activity behaviors; however, the majority of research to date has focused solely on TV watching [25]. Furthermore, boys and girls may have different preferences for screen-based activities and screen-based activities may differentially impact health behaviors across sex [26,27]. Thus, the primary aim of this study was to examine the associations among multiple screen-based activities (TV watching, videogame use, and computer use), physical activity, and dietary habits, in a sample of school-aged children in Guadalajara and Puerto Vallarta, Jalisco, and Mexico City, Mexico. The secondary aim of this study was to examine whether associations among screen-based activities (TV watching, videogame use, computer use), physical activity, and dietary habits differed by sex.

## 2. Materials and Methods

### 2.1. Participants

Schools in Guadalajara (*N* = 12) and Puerto Vallarta (*N* = 5) were selected for participation in this study by the State of Jalisco Secretary of Education. Schools in Mexico City (*N* = 20) were selected based on their participation in another obesity-related study. All children enrolled in third, fourth, and fifth grades at participating schools were eligible for participation in the current study. Information about the study and informed consent were sent home to parents with children. Parents interested in participating were asked to return the signed consent form to school the next day. At each school, children with a signed consent were guided through completing the child assent before they were enrolled in the study. All procedures and instruments used in this study were approved by the Institutional Review Board at the University of Houston.

### 2.2. Sociodemographic Data

A modified version of the fourth grade School Physical Activity and Nutrition (SPAN) survey was sent home with children for parents to complete. This tool has been used extensively for national surveillance on health behaviors including diet and physical activity in schoolchildren. The first section on the SPAN includes questions regarding demographic information on the child’s age, sex, and household income [28].

### 2.3. Screen Time

Three items on the SPAN instrument were used to assess leisure screen-based activities. These items assessed hours per day TV watching, playing video games, and recreational computer use. Parents reported the number of hours spent in screen-based activities using the following response options: 0 = My child does not watch TV, DVDs, or Movies/plays video games/uses a computer recreationally, 1 = Less than 1 h, 2 = 1 h, 3 = 2 h, 4 = 3 h, 5 = 4 h, 6 = 5 h, and 7 = 6 h or more. These SPAN items have reported acceptable validity and reliability for quantifying screen time [28,29].

### 2.4. Dietary Habits

Four items on SPAN instrument were also used to assess dietary intake the day prior to determine regular food behaviors and meal patterns [28]. Unhealthy and healthy dietary habits were assessed using three healthy food items: (1) fruit, (2) green salad, (3) vegetables; and three unhealthy food items: (4) high-fat meats (hamburgers, hot dogs, sausage, bacon, or ribs) (5) french fries or chips, and (6) sweets (rolls, doughnuts, cookies, brownies, pies, or cakes). Healthy food items were assigned a score of 0 if they were not consumed, 1 if they were consumed once, and 2 if they were consumed more than once during the previous day. Scores for the three healthy food items were summed to calculate a healthy dietary habits index score [30]. Unhealthy food items were assigned a score of 0 if they were not consumed, −1 if they were consumed once, and −2 if they were consumed more than once during the previous day. Scores for the three unhealthy food items were summed to calculate an unhealthy dietary habits index score [30].

### 2.5. Physical Activity

Four items on the SPAN instrument were used to assess physical activity [28]: (1) Last week, on which days did your child play outdoors for 30 min or more? (2) On most days, how does your child get to school? (3) During the past 12 months, on how many sports teams did your child play? (4) Does your child currently take part in any other organized physical activities or take lessons, such as martial arts, dance, gymnastics, soccer, baseball, or tennis? Items assessing participation in organized activities and sports were combined to form one variable.

### 2.6. Analyses

Variables were analyzed descriptively by calculating frequencies or means and standard deviations as appropriate. The *t*-tests and Chi-square tests were used to test differences by sex. Correlates of screen-based activities (TV watching, playing videogames, or computer use) were analyzed using separate multiple ordinal regression models. Since more than 40% of families did not report family income, we used two modeling strategies to account for missing data: listwise deletion and multiple imputation estimations. Multiple imputation was used to impute values for all of our missing analytical variables. Imputations were run using the Impute Missing Data Values function of SPSS version 22. A detailed description of this process has been previously published [31]. Both modeling strategies considered sex, age, active commuting to school, number of days playing outside, participation in organized physical activities or sports, healthy eating index and unhealthy eating index scores, as well as socioeconomic status as independent variables. To explore differences in correlates between boys and girls, we conducted separate regression models by sex. All models considered schools as the clustering variable. Final models were tested for specification error, goodness of fit and collinearity. Differences between unadjusted means and proportions and regression model estimates for main effects were considered significant if *p* < 0.05. All analyses were run using STATA v14 SE (Stata Corp, College Station, TX, USA). The multiple imputation sample was used to conduct the primary analysis presented in this study. The same analyses were conducted using data from the complete information sample and these findings are provided as Supplementary Information for comparative purposes in Tables S1–S3.

## 3. Results

Of the 1994 surveys originally collected, 874 had complete data for all outcomes of interest and all investigated correlates. Compared to the original sample (*N* = 1994), a higher proportion of children with complete (*N* = 874) data played videogames for less than 2 h/day (69.9 vs. 59.8%) and engaged in more active transportation (56.9% vs. 43.1%), while a smaller proportion of children with complete data participated in organized activities or sports (52.0% vs. 60.6%). A total of 51% of the sample were girls, with a mean age of 9.6 (±1.0) years across the total sample. About one-third (34.8%) spent more than 2 h per/day TV watching. However, fewer children exceeded two-hours per day using a computer (6.8%) or playing video games (5.8%). Boys spent more time playing videogames, participating in organized activities or sports, spent more days playing outdoors and had a higher unhealthy eating pattern score compared to girls (*p* < 0.05). Data were imputed for a total of 824 additional cases, yielding 1698 cases for multiple imputation estimations. No significant differences were found between the original sample and the multiple imputation sample, and similar distributions of demographic variables were observed in both samples. Further details on demographic characteristics are presented in Table 1 for the total sample and across sex.

Results of ordinal regression models for TV watching, video game use, and computer use among the total sample are shown in Table 2 and sex stratified models are shown in Table 3. Across the total sample, five correlates for computer use (three in girls and two in boys), five correlates for TV watching (three in boys and two in girls), and seven correlates for playing videogames (three in boys and four in girls) were identified. Common correlates across all three screen activities were participation in organized activities or sports, unhealthy dietary habits index score, and household income. No other measure of physical activity was correlated with the three screen activities.

### 3.1. TV Watching

Across the total sample, TV watching was positively associated with male sex, the unhealthy dietary habits index score, and household income, and negatively associated with participation in organized activities and sports and the healthy dietary habits index score. Sex stratified models showed that for boys, TV watching was positively associated with the unhealthy dietary habits index score and age and negatively associated with participation in organized activities and sports. For girls, TV watching was positively associated with the unhealthy dietary habits index score and negatively associated with the healthy dietary habits index score.

### 3.2. Video Game Use

Across the total sample, playing videogames was positively associated with male sex, age, participation in organized activities and sports, unhealthy eating pattern score, and higher household income, while negatively correlated with active transportation. Sex stratified models showed that for boys and girls, significant positive correlates for playing videogames were age, participation in organized activities and sports, and unhealthy dietary habits score. In contrast, playing videogames was positively associated with higher household income in girls.

### 3.3. Computer Use

Across the total sample, computer use was positively associated with age, participation in organized activities and sports, unhealthy dietary habits index score, and higher household income, while negatively correlated with active transportation. Sex stratified models showed that for boys and girls, computer use was positively associated with age and the unhealthy dietary habits index score. Additionally, computer use was positively associated with higher household income in girls.

## 4. Discussion

Given the rising rates of pediatric obesity in Mexican children, it is important to identify behaviors like screen-based activities that influence obesity-related behaviors including physical activity and diet. Therefore, this study examined the associations among screen-based activities (TV watching, videogame use, computer use), dietary habits, and physical activity in school-aged children in Guadalajara and Puerto Vallarta, Jalisco, and Mexico City, Mexico. We also examined differences in associations across sex. Our findings revealed that unhealthy dietary habits, household income, and participation in organized sports and teams were common correlates of all three screen-based activities examined. Information gained from these findings can be used to inform future intervention strategies aimed at reducing screen-use and improving diet and physical activity in an effort to address the growing obesity pandemic in Mexico.

The negative associations between all three screen-based activities and unhealthy dietary habits are consistent with other studies that have found that increased screen time is associated with poor dietary habits [13,17,20,22,32,33]. Screen-based activities can distract children from internal food cues, which can lead to unconscious overeating and eating in the absence of hunger [17,20]. The marketing of unhealthy snack foods is another potential underlying factor that may explain the relationship between screen use and poor dietary habits. Studies have demonstrated that food and beverage advertisements can lead to increased consumption of these items among children [19,33]. Mexico currently has government regulations to limit the advertisements of unhealthy food and beverages to children during peak viewing time among children [34,35]. However, there is little evidence to demonstrate that current regulations limit children’s exposure to unhealthy food advertisements or have an effect on diet behaviors in children [36]. For example, studies conducted in Mexico still observed a higher prevalence of commercials focused on food and beverages during children’s programming compared to programming for the general audience (25.8% vs. 15.4%) and these commercials typically focused on sugar drinks, sweets, and cereals with added sugar [37,38]. Stronger regulations are needed to limit advertising sponsored by corporations, limit the use of persuasive techniques (e.g., use of celebrities, free gifts), and regulate false health and nutrition claims made in advertisements [36]. Given the emergence of social media, online gaming, and mobile applications, these regulations should extend beyond television to include video games, computer games, and mobile phone applications [36,39,40].

Our study also demonstrated that all screen-based activities were positively associated with income. This was particularly true among girls, as video game and computer use remained positively correlated with income among girls when correlates were examined by sex. This is consistent with previous studies examining socioeconomic differences in screen-time among Mexican youth that also found that children from higher socioeconomic backgrounds reported higher access to screen devices including televisions, computers, and gaming systems, which can be costly [9,41].

While we observed that all screen-based activities were correlated with participation in organized activities or team sports, the direction of these relationships differed across screen-based activities. TV watching was negatively associated with organized activities or sports participation; however, when examined by sex, this relationship only remained significant among boys. These findings support that time spent watching TV can replace opportunities for engaging in physical activity, especially among boys [7,12]. In contrast to TV watching, video game and computer use were positively associated with participation in organized activities or sports teams. Income may be an underlying factor linking video game and computer use with organized activity or sports teams given that families who can afford video gaming or computer systems may also have the financial resources to enroll their child in organized activities and team sports, which can also be costly [42]. This may be especially true in Mexico, where regular schools have very short school days (4.5 h school days), precluding the inclusion of extra-curricular activities or sports as part of daily school activities [43]. There is also evidence to support that youth who play sports video games in particular, engage in more real-life sports, suggesting that video games may be self-socialization tools that impact beliefs and behaviors around real-life sports [44].

## 5. Strengths and Limitations

Strengths of this study include a sizable study population from three diverse geographic regions of Mexico. An additional strength includes the investigation of multiple screen-based activities and physical activity types. The majority of studies to date have focused on a singular screen-based activity while few have simultaneously examined multiple screen-based activities [25]. Last, this study also examined relationships among screen-based activities, diet, and physical activity behaviors by sex. There is a lack of studies that have captured differences in screen time use by sex [45]. While this study’s strengths have been noted, this study is not without limitations. Data on screen-based activities, diet, and physical activity were collected using the SPAN survey, which is a parent reported survey. Although parent reports of children’s behavior are a limitation, the SPAN survey is a reliable tool and was culturally adapted and pilot tested prior to use among this population. Another limitation includes the lack of complete data among the total sample. However, the use of multiple imputation was appropriate in this study [46], the same models were run for the complete population and multiple imputation sample, and there were no significant differences found between the samples.

## 6. Conclusions

Findings from this study demonstrate that screen-based activities are associated with unhealthy dietary habits, income, and participation in organized physical activity or sports teams. Boys engaged in more overall use of screen-based activities, and screen-based activities had a more deleterious impact on diet and physical activity behaviors among boys, particularly older boys, compared to girls. These findings suggest that there is a need to develop sex-specific strategies that acknowledge the differential use of screen-based activities across sex. Our findings also suggest the need for age-specific strategies that acknowledge differences in screen-based activities across age. More research is needed to understand income as an underlying factor that influences screen-based activities as different strategies for reducing screen-based activities may be needed for children from higher verses lower income households. Future research should also continue to identify correlates of screen-based activities to inform future strategies aimed at reducing screen-time among this high-risk population.

## Figures and Tables

**Table 1:** Descriptive characteristics of the sample.

	Multiple Imputation Sample (*n* = 1698)					
	Total Sample		Females		Males	
	% or Mean	95% CI	% or Mean	95% CI	% or Mean	95% CI
*N* (%)			53.5	(51.0, 56.0)	46.50	(44.0, 49.0)
Age (years, mean, SD)	9.6	(9.6, 9.6)	9.6	(9.6, 9.7)	9.6	(9.6, 9.7)
Television viewing (*n*, %)						
My child does not watch TV	4.0	(3.1, 4.9)	4.8	(3.4, 6.2)	3.1	(1.9, 4.4)
Less than 1 h	11.3	(9.8, 12.8)	12	(9.8, 14.1)	10.5	8.3, 12.7)
1 h	22.0	(20.0, 23.9)	22.7	(19.9, 25.4)	21.1	(18.3, 24.0)
2 h	28.7	(26.6, 30.9)	28.5	(25.5, 31.5)	29.0	(25.8, 32.3)
3 h	18.6	(16.7, 20.4)	18.5	(15.9, 21.1)	18.6	(15.8, 21.4)
4 h	8.5	(7.2, 9.9)	8.2	(6.4, 10.0)	9.0	(6.9, 11.0)
5 h	2.7	(1.9, 3.4)	2.3	(1.3, 3.2)	3.1	(1.9, 4.3)
6 h or more	4.2	(3.3, 5.2)	3.2	(2.0, 4.3)	5.5	(3.9, 7.1)
Computer use (*n*, %)						
My child does not use a computer	33.1	(30.9, 35.5)	34.3	(31.2, 37.4)	31.7	(28.4, 35.0)
Less than 1 h	25.2	(23.1, 27.3)	27.0	(24.0, 30.0)	23.2	(20.2, 26.2)
1 h	22.7	(20.7, 24.7)	21.2	(18.5, 23.9)	24.5	(21.4, 26.2)
2 h	11.1	(9.6, 12.6)	10.9	(8.8, 12.9)	11.3	(9.1, 13.5)
3 h	3.7	(2.8, 4.5)	3.4	(2.2, 4.6)	4.0	(2.5, 5.4)
4 h	1.6	(1.0, 2.2)	1.2	(0.5, 1.9)	2.0	(1.0, 3.0)
5 h	1.1	(0.6, 1.5)	1.1	(0.4, 1.9)	1.0	(2.6, 1.7)
6 h or more	1.6	(1.0, 2.2)	0.9	(0.2, 1.6)	2.4	(1.3, 3.5)
Playing videogames (*n*, %)						
My child does not play videogames	47.7	(45.3, 50.1)	62.3	(31.2, 37.4)	30.9	(27.6, 34.2)
Less than 1 h	17.6	(15.7, 19.4)	17.6	(15.1, 20.1)	17.5	(14.9, 20.2)
1 h	17.5	(15.7, 19.3)	11.4	(9.3, 13.5)	24.5	(21.5, 27.5)
2 h	8.3	(7.0, 9.7)	4.8	(3.4, 6.2)	12.4	(10.1, 14.8)
3 h	3.8	(2.9, 4.7)	2.1	(1.1, 3.0)	5.8	(4.2, 7.5)
4 h	2.1	(1.4, 2.7)	0.8	(1.7, 1.4)	3.5	(2.2, 4.9)
5 h	0.1	(0.0, 0.1)	0.4	(0.0, 0.1)	1.0	(0.3, 1.7)
6 h or more	0.2	(0.2, 0.3)	0.3	(0.0, 0.1)	4.2	(2.8, 5.6)
Active transportation (*n*, %)						
No	47.7	(45.2, 50.1)	47.2	(43.8, 50.5)	48.3	(44.7, 51.8)
Yes	52.3	(49.9, 54.7)	52.8	(49.5, 56.1)	51.7	(48.2, 55.3)
Days of outdoor play in the past week (mean, SD)	2.4	(2.2, 2.5)	2.1	(2.0, 2.7)	2.6	(2.5, 2.8)
Participation in organized activities or sports (*n*, %)						
No	44.7	(42.2, 47.1)	50.1	(46.3, 53.7)	38.4	(34.2, 42.6)
Yes	55.3	(52.9, 57.8)	49.9	(46.2, 53.7)	61.6	(57.4, 65.8)
Healthy food index score (mean, SD)	2.3	(2.2, 2.4)	2.3	(2.2, 2.4)	2.2	(2.1, 2.3)
Unhealthy food index score (mean, SD)	1.8	(1.7, 1.8)	1.7	(1.6, 1.8)	1.8	(1.7, 1.9)
Household income (*n*, %)						
Less than MXN 5000	49.0	(44.6, 53.3)	49.4	(44.3, 54.5)	48.5	(43.3, 53.6)
MXN 5000–MXN 999,999	33.1	(29.4, 36.9)	32.6	(27.2, 38.0)	33.8	(29.8, 37.8)
MXN 10,000 or more	17.9	(15.4, 20.4)	18.0	(15.2, 20.8)	17.8	(13.5, 22.0)

**Table 2:** Correlates among total sample for TV watching, video game, and computer use among Mexican school age children (*n* = 1698).

	TV Watching		Video Game Use		Computer Use	
	OR	(95% CI)	OR	[95% CI]	OR	(95% CI)
Sex						
Females	1.00	----	1.00	----	1.00	----
Males	1.27	**(1.02, 1.58)**	**3.87**	**(2.89, 5.20)**	1.15	(0.97, 1.36)
Age (years)	1.11	(1.00, 1.23)	**1.17**	**(1.08, 1.25)**	**1.33**	**(1.20, 1.46)**
Active transportation						
No	1.00	----	1.00	----	1.00	----
Yes	1.14	(0.97, 1.33)	**0.74**	**(0.58, 0.94)**	**0.68**	**(0.54, 0.87)**
Days of outdoor play	0.98	(0.95, 1.01)	1.00	(0.96, 1.05)	1.02	(0.99, 1.06)
Participation in organized activities or sports						
No	1.00	----	1.00	----	1.00	----
Yes	**0.80**	**(0.67, 0.94)**	**1.50**	**(1.19, 1.83)**	**1.25**	**(1.03, 1.51)**
Healthy food pattern (score)	**0.88**	**(0.81, 0.95)**	0.98	(0.90, 1.06)	0.97	(0.91, 1.04)
Unhealthy food pattern (score)	**1.28**	**(1.17, 1.41)**	**1.28**	**(1.15, 1.42)**	**1.17**	**(1.09, 1.25)**
Household income						
Less than MXN 5000	1.00	----	1.00	----	1.00	----
MXN 5000–MXN 999,999	**1.36**	**(1.11, 1.66)**	1.38	(0.92, 2.08)	1.25	(0.91, 1.70)
MXN 10,000 or more	1.36	(0.95, 1.96)	**1.95**	**(1.29, 2.93)**	**1.84**	**(1.33, 2.54)**
*p* trend	**0.035**		**0.01**			

OR = odds ratio; CI = confidence interval; numbers in bold indicate significant differences between boys and girls.

**Table 3:** Correlates for TV watching, video game, and computer use across sex in Mexican school age children (females = 907, males = 791).

	TV Watching				Video Game Use				Computer Use			
	Females		Males		Females		Males		Females		Males	
	OR	(95% CI)	OR	(95% CI)	OR	(95% CI)	OR	(95% CI)	OR	(95% CI)	OR	(95% CI)
Age (years)	1.06	(0.93, 1.21)	**1.17**	**(1.02, 1.34)**	**1.15**	**(1.02, 1.30)**	**1.21**	**(1.05, 1.38)**	**1.39**	**(1.22, 1.58)**	**1.27**	**(1.11, 1.45)**
Active transportation												
No	1.00	----	1.00	----	1.00	----	1.00	----	1.00	----	1.00	----
Yes	1.15	(0.91, 1.45)	1.14	(0.86, 1.52)	0.80	(0.62, 1.02)	0.72	(0.50, 1.03)	0.75	(0.53, 1.05)	0.61	(0.47, 0.80)
Days of outdoor play	1.00	(0.93, 1.08)	0.96	(0.92, 1.01)	0.98	(0.88, 1.09)	1.01	(0.94, 1.08)	1.03	(0.98, 1.08)	1.03	(0.96, 1.08)
Participation in organized activities or sports												
No	1.00	----	1.00	----	1.00	----	1.00	----	1.00	----	1.00	----
Yes	0.84	(0.68, 1.02)	**0.74**	**(0.55, 0.99)**	**1.65**	**(1.26, 2.17)**	**1.37**	**(1.14, 1.64)**	1.20	(0.93, 1.54)	1.25	(0.97, 1.75)
Healthy food index score	**0.85**	**(0.77, 0.95)**	0.91	(0.82, 1.01)	0.98	(0.88, 1.09)	0.98	(0.88, 1.08)	0.92	(0.82, 1.02)	1.03	(0.95, 1.11)
Unhealthy food index score	**1.26**	**(1.12, 1.40)**	**1.31**	**(1.11, 1.54)**	**1.19**	**(1.07, 1.31)**	**1.37**	**(1.14, 1.64)**	**1.16**	**(1.06, 1.28)**	**1.17**	**(1.05, 1.31)**
Household income												
Less than MXN 5000	1.00	----	1.00	----	1.00	----	1.00	----	1.00	----	1.00	----
MXN 5000–MXN 999,999	1.30	(0.96, 1.77)	**1.43**	**(1.01, 2.03)**	**1.59**	**(1.05, 2.39)**	1.25	(0.70, 2.24)	1.40	(0.88, 2.23)	1.09	(0.70, 1.91)
MXN 10,000 or more	1.36	(0.73, 2.53)	1.38	(0.74, 2.56)	**2.45**	**(1.54, 3.88)**	1.57	(0.77, 3.19)	**2.29**	**(1.46, 3.59)**	1.48	(0.90, 2.45)
*p* trend	0.213		0.171		**0.00**		0.19		**0.001**		0.18	

OR = odds ratio; CI = confidence interval; numbers in bold indicate significant differences between boys and girls.

## Data Availability

The data presented in this study are available on request from the corresponding author. The data are not publicly available due to ethical concerns regarding privacy.

## Supplementary Materials

The following are available online at https://www.mdpi.com/article/10.3390/ijerph18136788/s1, Table S1: Descriptive characteristics of complete information sample, Table S2: Correlates for TV watching, video game, and computer use among complete information sample, Table S3: Correlates for TV watching, video game, and computer use across sex in complete sample.
