# Supplementary material for: Associations between Screen-Based Activities, Physical Activity, and Dietary Habits in Mexican Schoolchildren
Source: Int J Environ Res Public Health. 2021 Jun 24;18(13):6788. doi: 10.3390/ijerph18136788 (PMC8297222; doi:10.3390/ijerph18136788)
Supplement: Supplementary file 1 [file ijerph-18-06788-s001.zip › ijerph-1257450-supplementary/Supplementary Materials/Table S1_Descriptive characteristics of complete information sample.pdf]

Table S1. Descriptive characteristics of complete information sample.

	Complete information sample (n=874)					
	Total sample		Girls		Boys	
	n or mean	% or SD	n or mean	% or SD	n or mean	% or SD
**N**			449	51.4	425	48.6
**Age (years)**	9.6	1.0	9.6	1.0	9.6	1.0
**Television viewing**						
**My child does not watch TV**	27	3.1	18	4.0	9	2.1
**Less than 1 hour**	73	8.4	37	8.2	36	8.5
**1 hour**	197	22.5	102	22.7	95	22.4
**2 hours**	274	31.4	140	31.2	134	31.5
**3 hours**	173	19.8	91	20.3	82	19.3
**4 hours**	87	10.0	44	9.8	43	10.1
**5 hours**	23	2.6	11	2.5	12	2.8
**6 hours or more**	20	2.3	6	1.3	14	3.3
**Computer use**						
**My child does not use a computer**	303	34.7	154	34.3	149	35.1
**Less than 1 hour**	233	26.7	134	29.8	99	23.3
**1 hour**	194	22.2	89	19.8	105	24.7
**2 hours**	89	10.2	52	11.6	37	8.7
**3 hours**	35	4.0	14	3.1	21	4.9
**4 hours**	11	1.3	3	0.7	8	1.9
**5 hours**	5	0.6	1	0.2	4	1.0
**6 hours or more**	4	0.5	2	0.5	2	0.5
**Playing videogames**						
**My child does not play videogames**	449	51.4	306	68.2	143	33.7
**Less than 1 hour**	151	17.3	71	15.8	80	18.8
**1 hour**	162	18.5	48	10.7	114	26.8
**2 hours**	62	7.1	15	3.3	47	11.1

**3 hours**	25	2.9	5	1.1	20	4.7
**4 hours**	11	1.3	1	0.2	10	2.4
**5 hours**	4	0.5	1	0.2	3	0.7
**6 hours or more**	10	1.1	2	0.5	8	1.9
**Active transportation**						
**No**	377	43.1	190	42.3	187	44.0
**Yes**	497	56.9	259	57.7	238	56.0
**Outdoor play in the past week (number of days)**	2.3	2.2	**2.1**	**2.0**	**2.6**	**2.3**
**Participation in organized activities or sports**						
**No**	420	48.1	243	**54.1**	**177**	**41.7**
**Yes**	454	52.0	206	**45.9**	**248**	**58.3**
**Healthy food index (score)**	2.3	1.4	2.3	1.4	2.3	1.4
**Unhealthy food index (score)**	1.7	1.1	1.7	1.0	1.8	1.2
**Household income**						
**Less than 5,000 MXN**	434	49.7	224	49.9	210	49.4
**$5,000 - $9,999.99 MXN**	294	33.6	147	32.7	147	34.6
**10,000 or more MXN**	146	16.7	78	17.4	68	16.0

Numbers in **bold** indicate significant differences between boys and girls.
[a] Estimations calculated using multiple imputation estimations.
