# Supplementary material for: Associations between Screen-Based Activities, Physical Activity, and Dietary Habits in Mexican Schoolchildren
Source: Int J Environ Res Public Health. 2021 Jun 24;18(13):6788. doi: 10.3390/ijerph18136788 (PMC8297222; doi:10.3390/ijerph18136788)
Supplement: Supplementary file 1 [file ijerph-18-06788-s001.zip › ijerph-1257450-supplementary/Supplementary Materials/Table S2_Correlates for TV watching, video game, & computer use across complete sample.pdf]

Table S2. Correlates for TV watching, video game, and computer use among complete information sample.

	TV Watching		Video Game Use		Computer Use	
	**OR**	**(95% CI)**	**OR**	**(95% CI)**	**OR**	**(95% CI)**
**Gender**						
**Girls**	1.00	----	1.00	----	1.00	----
**Boys**	1.20	(0.90, 1.60)	**4.68**	**(3.36, 6.51)**	1.13	(0.88, 1.48)
**Age (years)**	1.00	(0.87, 1.14)	**1.13**	**(1.02, 1.25)**	**1.24**	**(1.14, 1.41)**
**Active transportation**						
**No**	1.00	----	1.00	----	1.00	----
**Yes**	**1.36**	**(1.08, 1.73)**	0.80	(0.60, 1.08)	0.64	(0.48, 0.87)
**Days of outdoor play**	0.95	(0.89, 1.01)	0.98	(0.93, 1.03)	1.01	(0.95, 1.07)
**Participation in organized activities or sports**						
**No**	1.00	----	1.00	----	1.00	----
**Yes**	0.70	(0.53, 0.91)	1.28	(0.93, 1.78)	0.94	(0.73, 1.20)
**Healthy food pattern (score)**	**0.84**	**(0.77, 0.92)**	**0.86**	**(0.79, 0.94)**	0.95	(0.86, 1.05)
**Unhealthy food pattern (score)**	**1.29**	**(1.13, 1.48)**	**1.41**	**(1.22, 1.63)**	**1.17**	**(1.04, 1.32)**
**Household income**						
**Less than 5,000 MXN**	1.00	----	1.00	----	1.00	----
**$5,000 - $9,999.99 MXN**	**1.56**	**(1.20, 2.02)**	**1.94**	**(1.43, 2.64)**	**1.71**	**(1.21, 2.42)**
**10,000 or more MXN**	**1.75**	**(1.07, 2.87)**	**3.21**	**(2.14, 4.81)**	**3.49**	**(2.39, 5.12)**
p trend	**0.005**		**<0.001**		**<0.001**	

OR= Odds Ratio; CI= Confidence Interval

Numbers in **bold** indicate significant differences between boys and girls.

[a] Odds ratio calculated using an ordinal regression model.

[b] Odds ratio calculated using multiple imputation ordinal regression model.
