# Supplementary material for: Associations between Screen-Based Activities, Physical Activity, and Dietary Habits in Mexican Schoolchildren
Source: Int J Environ Res Public Health. 2021 Jun 24;18(13):6788. doi: 10.3390/ijerph18136788 (PMC8297222; doi:10.3390/ijerph18136788)
Supplement: Supplementary file 1 [file ijerph-18-06788-s001.zip › ijerph-1257450-supplementary/Supplementary Materials/Table S3_Correlates for TV watching, video game, & computer use across sex.pdf]

Table S3. Correlates for TV watching, video game, and computer use across sex in complete sample.

	Television Viewing				Video Game Use				Computer Use			
	Girls (n=449)		Boys (n=439)		Girls (n=907)		Boys (n=791)		Girls (n=449)		Boys (n=791)	
	OR	(95% CI)	OR	(95% CI)	OR	(95% CI)	OR	(95% CI)	OR	(95% CI)	OR	(95% CI)
**Gender**												
**Girls**												
**Boys**												
**Age (years)**	0.96	(0.83, 1.12)	1.04	(0.84, 1.28)	1.17	(0.94, 1.47)	1.13	(0.95, 1.34)	**1.26**	**(1.04, 1.52)**	**1.30**	**(1.13, 1.49)**
**Active transportation**												
**No**	1.00	----	1.00	----	1.00	----	1.00	----	1.00	----	1.00	----
**Yes**	**1.38**	**(1.02, 1.88)**	1.38	(0.91, 2.09)	0.78	(0.55, 1.11)	0.88	(0.62, 1.25)	0.74	(0.48, 1.12)	**0.56**	**(0.41, 0.77)**
**Days of outdoor play**	0.92	(0.83, 1.02)	0.97	(0.90, 1.05)	1.03	(0.96, 1.12)	0.97	(0.90, 1.04)	1.01	(0.94, 1.09)	1.01	(0.71, 1.45)
**Participation in organized activities or sports**												
**No**	1.00	----	1.00	----	1.00	----	1.00	----	1.00	----	1.00	----
**Yes**	0.81	(0.61, 1.09)	**0.58**	**(0.38, 0.88)**	**1.71**	**(1.21, 2.42)**	1.05	(0.70, 1.59)	0.90	(0.60, 1.27)	1.02	(0.71, 1.45)
**Healthy food index score**	**0.84**	**(0.73, 0.95)**	**0.85**	**(0.75, 0.96)**	0.89	(0.75, 1.06)	**0.83**	**(0.76, 0.91)**	0.94	(0.81, 1.10)	0.95	(0.84, 1.08)
**Unhealthy food index score**	**1.25**	**(1.06, 1.48)**	**1.36**	**(1.12, 1.65)**	**1.24**	**(1.03, 1.49)**	**1.53**	**(1.25, 1.88)**	**1.17**	**(1.04, 1.32)**	1.16	(0.94, 1.43)
**Household income**												
**Less than 5,000 MXN**	1.00	----	1.00	----	1.00	----	1.00	----	1.00	----	1.00	----
**$5,000 - $9,999.99 MXN**	1.38	(0.90, 2.11)	**1.77**	**(1.23, 2.56)**	**2.39**	**(1.40, 4.08)**	**1.78**	**(1.31, 2.43)**	**1.71**	**(1.21, 2.42)**	**2.00**	**(1.32, 3.54)**
**10,000 or more MXN**	**1.94**	**(1.10, 3.42)**	1.42	(0.70, 2.88)	**4.43**	**(2.26, 8.7)**	**2.43**	**(1.49, 4.00)**	**3.49**	**(2.39, 5.12)**	**5.12**	**(3.07, 8.54)**
p trend	**0.018**		0.080		**<0.001**		**<0.001**		**<0.001**		**<0.001**	

OR= Odds Ratio; CI= Confidence Interval

Numbers in **bold** indicate significant differences between boys and girls.

[a] Odds ratio calculated using an ordinal regression model.

[b] Odds ratio calculated using multiple imputation ordinal regression model.
